# Shifting between roles of a customer and a seller – patients’ experiences of the encounter with primary care physicians when suspicions of cancer exist

**DOI:** 10.1080/17482631.2021.2001894

**Published:** 2021-11-17

**Authors:** Cecilia Hultstrand, Anna-Britt Coe, Mikael Lilja, Senada Hajdarevic

**Affiliations:** a Department of Nursing, Umeå University, Umeå, Sweden; b Department of Public Health and Clinical Medicine, Family Medicine, Umeå University, Umeå, Sweden; c Department of Sociology, Umeå University, Umeå, Sweden; d Department of Public Health and Clinical Medicine, Unit of Research, Education, and Development, Östersund Hospital, Umeå University, Umeå, Sweden

**Keywords:** Cancer, symptoms, seeking care, primary healthcare, patients’ experiences, encounters, standardization, CPP

## Abstract

**Purpose:**

Sweden has tried to speed up the process of early cancer detection by standardization of care. This increased focus on early cancer detection provides people with a conflicting norm regarding the importance of recognizing possible cancer symptoms and the responsibility of not delaying seeking care.

Based on existing norms about patients’ responsibility and care seeking, this study explores how patients experience encounters with primary care physicians when they seek care for symptoms potentially indicating cancer.

**Methods:**

Thirteen semi-structured interviews were conducted with patients receiving care for symptoms indicative of cancer in one county in northern Sweden. Data was analysed with thematic analysis.

**Results:**

The common notion of describing patients as customers in a healthcare context does not sufficiently capture all aspects of what counts as being a person seeking care. Instead, people interacting with primary care face a twofold role in where they are required to take the role not only of *customer* but also of *seller*. Consequently, people shift between these two roles in order to legitimize their care seeking.

**Conclusions:**

Standardization oversimplifies the complexity underlying patients’ experience of care seeking and interaction with healthcare. Hence, healthcare must acknowledge the individual person within a standardized system.

## Introduction

The increased focus in society on early detection of cancer provides people with a new norm regarding the importance of recognizing possible cancer symptoms and not delaying care seeking (e.g., Quaife et al., 2013; Simon et al., 2010).

This norm places the responsibility of seeking healthcare in a timely manner on people (Robb et al., 2009; Simon et al., 2010). When people do not recognize alarm symptoms and thereby do not seek care in time, they are often described as delaying their cancer diagnosis (Quaife et al., 2013) and therefore several countries have tried to speed up the process of early cancer detection by standardization of care. Meanwhile, in many settings including Sweden, another perceived norm exists, to avoid seeking care unless necessary (Hajdarevic, 2012; Whitaker et al., 2015). Additionally, following the norm of being a “good citizen” people report hesitating to seek care in order to not waste healthcare resources, including their doctor’s time (Andersen et al., 2011; Whitaker et al., 2015). These norms appear to underlie a growing ambivalence found in people's interaction with the healthcare system (Ziebland et al., 2019), and include conflicting responsibilities related to care seeking. On the one hand, people should not delay their care seeking, on the other hand, people should not consume healthcare resources unnecessarily, which places people in an ambivalent position. Thus, there is a paradox related to care-seeking, which we understand as a conflicting norm. This paper explores these conflicting norms by focusing on patients’ experiences of encounters with primary care physicians (PCPs) where standardized cancer patient pathways (CPPs) have been adopted. We try to understand how these norms take shape during patients’ experiences with healthcare. Previous literature describes the decision to seek care as a complex process for the individuals, encompassing internal negotiations of the seriousness of the experienced sensations and symptoms (e.g., Hajdarevic et al., 2010, 2011; Offersen et al., 2016; Macartney et al., 2017). However, previous research has focused less about patients’ experiences of conflicting norms during interactions with healthcare; in this article, we use the case of patients seeking care for symptoms potentially indicating cancer.

### Conflicting norms in socially constructed healthcare systems

Following a social constructionist paradigm, we understand phenomena such as health and healthcare as created from and within human interactions. Health and illness is defined not merely through biomedical conceptualizations, but also as it relates to our social world and to our interpretation of our experiences situated within our cultural context (Burr, 2015).

Similarly, the healthcare system is socially constructed, shaped by joint views and shared meanings of, for example, how to use healthcare resources appropriately (Kleinman, 1980). The healthcare system functions by legitimated social norms that influence how people react to sickness and how they perceive the system itself and its resources. Hence, healthcare is a part of, and not isolated, from society (Kleinman, 1980).

According to Ziebland et al., people face contradictory norms when interacting with the healthcare system, managing these contradictions and ambivalences makes them good citizens. They give various examples of contradictory norms such as being observant to bodily changes, but not to hypochondriac levels; respecting physicians’ time, but not delaying seeking care; being observant to symptom awareness campaigns, but not seeking care unnecessarily; and accepting physicians’ reassurance, but listening to their own body and be willing to challenge the given advice. Additionally, people are expected to trust experts, but recognize and accept personal responsibility for the own health (Ziebland et al., 2019). We see these contradictions as stemming from a broader problem of two conflicting norms in the medical sphere, namely that responsible citizens recognize and seek healthcare for serious symptoms, but at the same time not waste the doctor’s time and resources (e.g., Freund, 2003). The attributes of a good citizen can be aligned with the responsibilities and privileges related to the ´sick role´ described by Parsons (1964). The ´sick role´ allows deviation from societal norm of being a healthy member, but entails the expectation of seeking care and cooperate with medical experts in order to exit the ´sick role´. These expectations and conflicting norms that people face when interacting with healthcare are understood as social constructs. As Burr (2015) describes, peoples’ understanding and knowledge, e.g., norms related to healthcare seeking, are constructed though their shared version of reality.

During recent decades, many countries including Sweden have undertaken significant changes to the management of healthcare systems with the goal of increasing effectiveness and ensuring quality of care (Mol, 2011). These changes are partly borrowed from the field economics, namely that by giving patients the possibility to choose their care providers will stimulate both care provision and providers’ performance, which in turn will improve quality of and access to care. Central to this management approach is the definition of patients as customers, which implies individualized tailored services. Meanwhile, healthcare (services) is increasingly adopting standardization, which implies that healthcare follow predetermined routines more like a cookie cutter. Despite aims to improve quality of, and access to care, this mis-match might counteract such aims (Mol, 2011).

Patients have left the discursive role of being passive patients and instead entered an active role, often described as customers, consumers, or clients, placing responsibility on them to be prepared for the encounter with healthcare (Hartzband & Groopman, 2011; Mclaughlin, 2009; Mol, 2011; Mol et al., 2010; Nettleton, 2013). The active role entails involvement and engagement in the own healthcare process (Boyer & Lutfey, 2010; Michailakis & Schirmer, 2010; Nordgren, 2008); thus, the medical encounter is nowadays often portrayed as a service meeting rather as a medical consultation (Nordgren, 2008). Since patients have gained power due to the increased availability of medical information (especially online), they have become “informed consumers” rather than “acquiescent patients” (Jutel, 2011).

Nonetheless, patients and physicians are dependent on each other during the encounter (Hultstrand, et al., 2020a), and the changed patient role has influenced the interaction between the patient and the healthcare system, including how access to healthcare services is perceived. Besides the big concerns with a potential illness when seeking care, patients face challenges with having to legitimize their complaints and properly interact with their physician and the healthcare system (Andersen et al., 2011).

Additionally, a stronger emphasis on early detections of cancer forces the process of what is being considered as symptoms to expand and subdivide, and in turn change the moral and social value placed on bodily sensations (Andersen, 2017). Such normative changes could be expected to reinforce demands upon patients to explain specify and justify when interacting with healthcare services when suspecting serious illness as cancer.

### Early detection of cancer in primary care

Patients suspecting serious illness often have the first contact with primary care (Rubin et al., 2015). In Sweden, primary care has become even more important since the introduction of standardized cancer patient pathways (CPPs) in 2015. The goal of CPPs is to shorten the time interval between well-founded suspicion of cancer, i.e., presence of alarm symptoms and/or signs of suspected malignancy and start of treatment (Wilkens et al., 2016). The initial assessment of such symptoms is typically performed by nurses and physicians in primary care and can end up in either a fast track (CPP) or a routine care procedure.

Patients seeking care present their experienced bodily sensations to a primary care nurse or a PCP during an encounter. This can be a very difficult task for the patient, especially if the experienced sensations are vague or diffuse (Offersen et al., 2016), which most symptoms presented in primary care area (Ingeman et al., 2015). Symptoms potentially indicating cancer, i.e., alarm symptoms, have gained a powerful symbolic value both for the CPP as an entrance to a fast track and in terms of being, as Andersen (2017) portrays, “abstract-able forms of body-knowledge”, which might function as a driving force for people to seek care (Andersen, 2017). These alarm symptoms of cancer are, however, common among the general population (Ingebrigtsen et al., 2013; Winstanley et al., 2016), which make it even more complex to suspect or exclude cancer as well as a single alarm symptom is seldom connected to a cancer disease (Ewing et al., 2016; Ingebrigtsen et al., 2013; Lyratzopoulos et al., 2015; Rubin et al., 2015). Consequently, patients presenting with vague and diffuse sensations often experience it as more problematic to legitimate their care seeking, in comparison with those patients who seek care for a more well-defined chief complaint (Andersen et al., 2015).

Diagnosing cancer is a multistep process, influenced by factors related to the patient, the PCP and the healthcare system. The encounter between patient and PCP constitutes one process where opportunities of diagnosing cancer might risk to be missed, due to, for example, communication difficulties, patients’ failure of presenting symptoms and physician’s failure related to triaging and examining (Lyratzopoulos et al., 2015; Singh et al., 2013). Moreover, the way patients present their experienced sensations can influence their diagnostic care trajectory (Seibaek et al., 2011).

### Aim

Based on existing norms about patients’ responsibility and care seeking, this study aims to explore how patients experience encounters with primary care physicians when they seek care for symptoms potentially indicating cancer. The following research questions are adopted: What expectations and perceived responsibility do patients have on themselves and on their primary care physician, in the encounter? How do patients perceive their role in the interaction with their primary care physician?

## Methods

### Context description

Healthcare in Sweden is publicly available, tax funded and decentralized. Primary healthcare centres (PHCs) are publicly funded, though sometimes provided by private entrepreneurs. Inhabitants choose the PHC they want to be listed at, i.e., from which PHC they want to receive healthcare services. The most common way to make an appointment at a PHC is to call the PHC or to use the web-based platform 1177.se. The Swedish Healthcare Guarantee (*Vårdgaranti in Swedish*) ensures a response from the PHC by telephone or video call, at minimum, the same day. Further, if it is medically motivated, the Guarantee ensures that a person will be offered a medical assessment by a primary care nurse or a PCP within 3 days from contacting their PHC. Additionally, on referral, an appointment with specialist care is guaranteed within 90 days (Healt and Medical Act 2017:80). However, following recommendations from the introduction of CPPs, the numbers of days for a specialist appointment has been greatly reduced and are specified in each care pathway for each cancer diagnosis. For example, for a patient referred into a CPP based on a well-founded suspicion of colorectal cancer, the number of days from referral from primary care to the examination in specialist care is specified to 10 days (Regional Cancer Center).

### Participants and recruitment strategy

This study is based on material from individual interviews, with patients seeking care for sensations/symptoms potentially indicating cancer. Hence, inclusion criteria were patients (≥18 years) who have sought care at publicly available primary healthcare centres in one region in northern Sweden for sensation/symptoms that could indicate cancer or had worries about cancer. The participants’ age ranged from 41 to 82 years (mean 65.5), and Table I provides an overview of the sensations/symptoms that the study participants sought care for.

**Table I.** Participants’ experienced sensations/symptoms

Symptom	Men	Women
Rectal bleeding	i	ii
Lump	i	ii
Skin lesion	i	
Fatigue		ii
Stomach complaint	i	ii
Cough	i	

Prior to the interviews, all patients participated in an observational study Hultstrand, et al., 2020a), where the first and/or last author observed their encounter with their PCP. Participants were consecutively invited to participate in the observational study, and we observed patients who sought care at four PHCs in one county in northern Sweden, for bodily sensations/symptoms potentially indicating cancer. Patients were initially briefly informed by the healthcare personnel about the study when they called the PHC to make an appointment, but after the appointment had been made. Thereafter, the researchers (CH, and/or SH) met up with the patients in the waiting room before their booked appointment at the PHC. The researcher(s) provided the patient with oral and written information about the study and invited the patient to ask questions. We informed the patients that we aimed to explore their first encounter and the entry into care, the word cancer was not mentioned. Also, patients were informed that regardless if they wanted to participate or not, it would not affect their given care. In total, 18 patients were observed in the previous observational study, of whom 16 were invited to participate in this interview study. Two patients were not asked to participate because we failed with getting in contact with one of them, and we perceived the other to be in too poor health. In total, 13 patients accepted, and are thus included in this study.

### Data collection

Data consists of material from 13 semi-structured interviews with patients, conducted by the first author CH (n = 11) and last author SH (n = 2), three to 30 days (median 6 days) after their participation in the observational study Hultstrand, et al., 2020a), at a time and place chosen by the participants. All interviews were audio recorded and transcribed verbatim, which resulted in 185 pages with text. The interviews lasted between 15 and 49 minutes (median 37 minutes), and the word “cancer” was not used by the interviewer unless the interviewee mentioned it.

The interviews followed an interview guide with open-ended questions, such as “Can you describe the expectations you had before the encounter?”, “Can you describe how it was to tell your doctor about why you were seeking care?” and “How do you experience that the doctor listened and understood you reasons for seeking care?”

### Ethical considerations

Written informed consent was obtained from all participants and ethical approval was granted from the regional ethical review board (Dnr. 2017–296–31M; 2018–242–32M). Participation was on voluntary basis, meaning that participants could withdraw from participation at any time.

### Data analysis

The authors who analysed the material collaborated in an interdisciplinary research team, consisting of knowledge from the fields of public health (CH), sociology (ABC), family medicine (ML), and nursing (SH). We have utilized social constructionism as our ontological and epistemological stand point, which has guided our data gathering, analysis and interpretation. For example, we view data as a social construct derived from the interaction between the interviewer and the interviewee.

The data were analysed following thematic analysis in order to identify patterns of meaning across a set of data and explore features related to participants’ lived experiences (Braun & Clarke, 2006; Clarke & Braun, 2017). Following social constructionism, our analysis went beyond participants’ manifest or explicit statements to focus on latent meanings that were developed in the interview material. Specifically, we strived to understand the underlying assumptions and notions, and interpret these within the sociocultural healthcare context (Braun & Clarke, 2006). Initially, we engaged in repeated readings of our interview transcripts and wrote down emerging ideas. Ideas that early emerged were related to difficulties patients face when they try to verbalize their sensations, as well as their worries about seeking care unnecessarily, which helped us develop a more thorough understanding of the data. Second, we conducted initial coding by going through transcripts line by line, and assigning codes to the text. Some codes used words that the participants expressed, and others were our own word following our understanding of the underlying meanings. This step was performed by CH with continuous discussion with ABC, SH, and ML. Third, we search for potential themes and sub-themes by clustering codes that we interpreted as belonging together. This was performed when the majority of transcripts were analysed. Themes were created by clustering sub-themes that were related to each other. Lastly, we reviewed and revised our themes, which include the process of going back to our extract and transcripts to ensure that all codes building up to a specific theme are appearing from a coherent pattern, as well as naming our final themes (Braun & Clarke, 2006). During this final step, based on the underlying meanings of encounters in primary care as a part of healthcare services, we also theorized our results using the prominent attributes of customer and seller. An example of the coding process, including sub-themes for one theme, is presented in Table II. All themes were discussed among all authors until consensus was reached. The software program MAXQDA version 2018.2 was used for coding, managing and analysis.

**Table II.** Example from the analysis process: transcript, initial coding, sub-theme and theme

Text from transcript	Initial coding	Sub-theme	Theme
*I can’t, I find it difficult to describe how I feel and what it is, because really don’t know what’s wrong …*	Describing how I feel is difficult, Not knowing what’s wrong	Presenting experienced bodily sensations is challenging	**Being prepared to present bodily sensations**
*… it feels like it’s now or never, and to remember everything, to describe, well to remember everything that has happened. I have to perform a lot of information in a short period of time … I need to keep track on what I want to say.*	Seizing the opportunity, Remembering is challenging, Having to perform	Feeling demands on myself when presenting bodily sensations	

## Results

In light of the contemporary development of the healthcare organization, people are often treated as customers. However, based on our results, describing people seeking care as customers does not successfully capture all aspects of seeking care. Rather, people interacting with healthcare face a twofold role, where they are required to take the role not only of a *customer* but also of a *seller*.

We found this dual role across and within the three themes that resulted from our analysis: *Being prepared to present bodily sensations, Expecting a clear plan as an acknowledgement*, and *Receiving a straight pathway—or continuing to deal with uncertainties*. The first theme depicts patients’ perceived demands upon themselves when seeking care, while the second theme depicts their expectations when seeking primary healthcare. Lastly, the third theme depicts patient’s experiences of being either referred into standardized fast routine or being offered the non-standardized alternative (traditional procedures).

### Being prepared to present bodily sensations

This theme depicts patients’ perceived demands on themselves when presenting their experienced bodily changes to their PCP in order to get appropriate care. Challenges and responsibilities that patients face when they present or “sell” their sensations and symptoms to their PCP are illuminated, which resemble the attributes of a *seller* on the market. Firstly, the theme illuminates patients’ difficulties and challenges with verbalizing the experienced bodily sensations potentially indicating cancer. Patients described that seeking care for symptoms, potentially indicating cancer is rather special, saying that it is easier to seek care for example, an ear infection, or a symptom that is concrete and visible by eye gaze, compared to, what patients often referred to, as “mysterious” and difficult to interpret and define as symptoms. Patients talked in terms of being a case for a detective, meaning that the PCP had the puzzling task of being the detective, trying to figure out what was wrong in the patient’s body. Some patients described that they experienced a feeling of something not right in their body, which was described as difficult to put into words to make the PCP understand and to make the PCP recognize the seriousness of the patients’ condition. Interestingly, patients who experienced more vague and diffuse sensations, e.g., tiredness, and were not referred into a CPP, expressed it as particularly challenging and demanding to present their bodily sensations precisely and in detail. As one informant conveyed:

*it’s difficult to explain how I feel, how it feels in my body … If I have broken an arm it is very easy, but that which is not visible on the outside [of the body] how does one explain that, so that the doctor feels that this is seriously, this is not made up … (Interviewee C, fatigue, not CPP)*

Second, the theme depicts that how the sensations are verbalized is perceived to affect the received care and thus patients' care trajectories. Returning to the metaphor of being a case for a detective, patients expressed having doubts of what and how to express their reasons for care seeking to their PCP. When the patterns of bodily sensations were not clearly identified and not recognized, it was experienced as challenging since they could not present it as a symptom in an assuring way. These kinds of feelings were most prominent among those patients who were not referred into a CPP. Some patients described worries about how they had presented and worried that they might have failed with their presentations or not have presented in an optimal way to make the PCP really understand what was bothering them. Patients talked about the encounter as the one chance to deliver or “sell”, “the right information” to the PCP, to make the PCP understand the patients’ health situation and thus make and appropriate assessment and a plan for future actions in the patients' care trajectory. Consequently, patients enter the role as a *seller* during encounters, which often entail feeling pressure and that much are at stake during the encounter, in terms of seizing the opportunity with the PCP and do their best to present their experienced bodily changes. Thus, patients described that what they said during the short time of encounter would affect their future care and that what they perceived as a good presentation (i.e., clear, structured, assuring) would assist their detective, i.e., doctor to find the solution.

Lastly, the theme depicts the perceived need of being prepared for the encounter to be able to present the experienced sensations maximally well without jeopardizing the opportunity, or in other words embodying the role of a *seller* trying to “sell in the sensation”. Hence, patients expressed feeling that their future care was dependent on how they delivered their presentation of their reasons for care seeking, i.e., their experienced bodily sensations and symptoms, even when these were described as mysterious or diffuse. Some patients described that they had prepared themselves for the encounter, by for example, making a list with symptoms to describe or in other ways mentally prepare their presentations.

*the person [PCP] doesn’t know me I meet her very briefly, she has no idea about my medical history or things that usually bother me, that’s why I need to have my little power point presentation ready in my head (Interviewee F, stomach complaint, not CPP)*

Limited time with the PCP was additionally one factor that patients described made them feel under pressure and that they had to deliver much adequate information during a short period of time, especially if they met a PCP they had never met before. Also, patients expressed that they felt that their presentation to the PCP need to be rather quick and interesting to listen to, meaning that they did not want their PCP to become bored or tired of them and their presentations. Patients also perceived themselves as nagging if they repeated their stories when talking to their PCP. As one informant stated:

*… well you don’t want to trouble [the PCP] for too long, you feel that it [presentation] should go fast, I have experienced that before. Don’t babble for such awfully long time … (Interviewee M, stomach complaint, not CPP)*

In sum, patients perceive a need to be prepared to deliver and perform detailed and adequate presentations of their symptoms that are indicative of cancer when encountering healthcare, which are attributes pertinent to those of a *seller* on the market.

### Expecting a clear plan as an acknowledgement

This theme depicts patients’ expectations of being referred beyond primary care and given a clear plan for their healthcare trajectory. Patients’ wants, demands and requests to move forward in the healthcare system chain are illuminated, which resembles attributes of a *customer* on the market that can pick and choose between goods and services.

Firstly, the theme illuminates that patients want and wish for a plan for the future as a result of their encounter with the PCP. Regardless of whether the patients were referred into a CPP or not, patients described that they want a plan for the close future, they expressed that they need information about what will happen next and that there is a way forward in their care trajectory. Knowing that they are on their way to be further examined and investigated, after the encounter in primary care, made them feel secure since such plan made them feel that they were in good hands, understood and taken seriously. It was important for patients that their PCP told them explicitly about the plan that their PCP had set for them, meaning that their sensations and worries were taken seriously, and they know what was going to happen next.

*… was in that situation where cancer can’t be ruled out, and you know that there is a way forward to go and that is to examine the breast first, that is, with biopsy and x-ray before you can 100% ensure what it is, and given my ages, well yes … (Interviewee J, lump, CPP)*

Second, the theme depicts that patients expect to be referred forward in the healthcare system chain after the visit in primary care; thus, primary care is in this theme often perceived as only the entrance into the healthcare system. This resembles a *customer* on the market, thus, we theorize that patients embody the role as customers when they express expectations of being referred to secondary care, since these expectations are related to “products” patients (customers) want to “consume” or “buy”. To initially seek care at PHCs was although described as the “right way” into the healthcare system and the way that you, as a good citizen, are supposed to take before being able to be referred and to receive further care from specialist clinics. Patients described that they were expecting further referrals, and that they perceive primary healthcare as only the entrance, since the care they considered that they need is not available and cannot be performed at PHCs. Thus, patients expressed that they expected that their care trajectory start at the PHC and then goes on to secondary care.

*… I thought that there is nothing anyone can do at the primary healthcare center, that I’m completely convinced about, so there had to be something beyond that, I was quite clear about that … Well, that is healthcare in a nutshell as it’s organized today, you never get anything altogether in one place. (Interviewee E, lump, not CPP)*

Furthermore, patients described that they want and expect to be referred to further examinations and investigations, in particular, they had expectations of being referred to specialist clinics. Such referral was explained to generate feelings of trust for the PCP, and made patients feel that they were being taken seriously. As one informant put it:

Interviewer “*you mentioned being taken seriously, what should the doctor do to make you feel that way*?”

Informant “*Uuum, well, I think it is about, well ´we can refer you to this person who knows about these things´, well, I want more specialist examinations I think*.” (Interviewee F, stomach complaint, not CPP)

As a customer on the market, patients sometimes verbalized specific requests, which exemplifies how patients embody the role as a customer when trying to ensure that they themselves would move forward in healthcare system chain. As one informant described:

*My trust that the physician is the one who takes me forward in the healthcare system chain is quite small, I have to do that myself, that’s how it feels. (Interviewee F, stomach complaint, not CPP)*

In sum, patients have expectations related to their care trajectories, such as being informed and being referred above and beyond primary care. Thus, as a customer on the market, patients verbalize their perceived wants and needs in order to receive the care that they expect.

### Receiving a straight pathway—or continuing to deal with uncertainties

This theme depicts patients' experiences of being referred into a standardized cancer patient pathway (CPP), or to the contrary, being offered a non-standardized alternative. Thus, this theme illuminates experiences of different trajectories, thus captures attributes that resembles a *customer’s* experiences of different services. The first part of this theme highlights experiences from those patients who were referred into a CPP (n = 4) since they had symptoms that were recognized by the PCP as alarming and thus matching the criteria for CPP. First, the theme illuminates that a fast track system, that a CPP offers, reduces painful uncertainty and vacuum feelings, thereby reducing painful feelings associated with long waiting for the next step. It thereby minimized anxieties and worries that can result from long waiting times. Patients described the time when being faced with a possible cancer diagnosis as very strenuous, characterized by much worries and anxieties. A referral through a fast track system, such as CPP, was emphasized as beneficial even if it could end in a cancer diagnosis. This indicates a need for clarity regarding what is beyond the symptoms and the possible solutions. Rather than a painful void where one suspects something serious, the study participants wanted to proceed further to find out the answers. This reveals a readiness to go through the process of investigation in order to minimize unnecessary worries associated with long intervals between the encounter in primary care and investigation and potential treatment. Thus, CPPs were described as reducing anxieties both in those cases where cancer could be detected and because treatment could begin rapidly, as well as in those cases where cancer could be ruled out because it would relieve worries.

Interviewer “*So next step is to do this mammography and biopsy, how does that feel for you?*”

Informant “*Yes, it feels great, and that it went so fast, it surprises me that it only happened in a few days, so I’m very happy with that, because it is mentally difficult to wait and wait for something that maybe takes weeks, because the worries constantly gnaws, and okay if you then get a cancer diagnosis, but if you don’t, then you have been worried for nothing, it is so unnecessary*.” (Interviewee J, lump, CPP)

Secondly, the theme depicts the positive understandings of the fast track offered by CPPs. Patients referred into a CPP understood the importance of a timely diagnosis and described the expectation to be referred forward in the healthcare system chain. Therefore, they valued their referral into the fast track system that CPPs intend to be, thus like a *customer* on the market, patients expressed satisfaction with the service they received. Nevertheless, the interviewed patients were not familiar with the concept of CPPs, they had not heard about it before; however, they expressed that the information that their case would be handled much faster was enough for them to know.

Lastly, the theme depicts patients’ satisfaction with their symptom presentation as they felt that their verbalized experienced bodily sensations were taken seriously and confirmed when being referred into a CPP, which illuminates that they successfully managed their twofold role, as both *seller* and *customer*. Even though patients experiencing symptoms potentially indicating cancer described that they expected to be referred to secondary care, some patients were surprised by the short time interval between their visits in primary care and their visits for specialist examination. This was highly valued and appreciated and resembles attributes of a satisfied *customer*. Moreover, patients who were referred into a CPP expressed that they felt like they and their reasons for care seeking were taken seriously.

*… he explained that he was going to write a referral to, it was called like fast something [referring to CPP], and that I think feels good, and it felt good when I left [the PHC] that it [the health issues] was taken seriously. (Interviewee L, rectal bleeding, CPP)*

The second part of this theme highlights experiences from those patients (n = 9) where referral to standardized pathways was not applicable, i.e., they were offered usual care, a non-standardized route. These include experiences such as dealing with bodily sensations and seeking care for these sensations and symptoms that are not easy to define nor recognize, as well as difficult to identify in the fast track route criteria. These non-standardized alternatives include a range of different examinations and testing, either at the PHC or routine referrals to secondary care, such as ultrasound. Others were given medication and scheduled for follow-up in 2 weeks.

Firstly, as we learned in our previous theme, when patients seek primary healthcare they expect a clear plan for their care trajectory, as well as to be referred above and beyond primary care. However, in this theme, we learn that this is not always the case. Patients not referred into a CPP expressed not knowing what the next step of their healthcare trajectory would be, which caused feelings of uncertainty and sometimes frustration. It could be related to a responsible *customer* who due to a perceived problem needs and seeks helpful service but not always receives it, since the provider cannot specifically (medically) define the problem and thereby not able to offer a specific service. At the same time, patients did recognize the examination of the human body as complex and difficult, and did not blame the PCP for not having an answer to their health complaint. As one informant put it

*I don’t know what the proceeding will be, if the tests look better, or if it point to something special, or if one can do anything about it, or is it just the way it is, I don’t know. It is like, I want a proceeding and in some way, an explanation is always nice, but I understand that it might not always exist, and that is not his fault. (Interviewee H, fatigue, not CPP)*

However, when patients' wants and desires of being referred beyond primary care were fulfilled, patients expressed satisfaction with their appointment at the PHC.

*Interviewer* *: What was it that made you satisfied?*

*Informant* *: That it would continue to be an investigation of my problem, he [the PCP] can’t do anything more, he did what he could.”* (Interviewee A, lump, not CPP)

Lastly, this theme depicts patients' need and desire to be informed about the whole process of examining and explaining their symptoms, including what test is being ordered and why, and when they can expect to hear back from the PCP with, for example, test results. In contrast to the information provided in conjunction with a CPP referral, information provided to the patients not referred into a CPP was perceived as insufficient and inadequate. Patients described the lack of answers and explanations as contributing to feelings of uncertainty and dissatisfaction. The information that patients expressed a desire for includes information about the probe. They wanted the PCP to talk about what was happening and why during the probe. Being informed about the whole process and procedures made patients satisfied, feel taken care of, and made them believe that their health problem was on its way of being solved, as one informant put it

*The PCP explained a little bit of this and that, and that he will take a picture and send it [to specialist care], he explained the whole process, so that was good. It was a very good appointment. (Interviewee I, Skin lesion, Not CPP)*

In sum, this theme depicts the experiences of being referred into this fast track, which contributed with a feeling of being taken care of by the healthcare system, despite that they were aware of that this route could imply a cancer diagnosis. It also depicts experiences of challenges when seeking care for sensations and symptoms that were more demanding to present and were not matching the criteria for the fast track route as CPP, and thereby continuing to deal with uncertainties and repeated contact with healthcare. However, to find a solution was always the main goal. When patients were informed and promised that their case would proceed in one way or another, this was in some way contributing to feelings of being taken care of, even though they still have to deal with some level of uncertainty.

## Discussion

In view of the conflicting norms that people face when interacting with the healthcare system outlined earlier in this paper, our results suggest that patients experience a twofold role during encounters with physicians in primary care. Patients in our study experienced the responsibility to act not only as a *customer* but also as a *seller*. This illustrates how conflicting norms of seeking care in a timely manner without wasting time and resources, embedded in the healthcare system, shape patients’ experiences with primary care and put additional responsibility on them to manage these norms. Our results offer a new understanding of the commonplace notions of patients as customers, consumers, or clients (Hartzband & Groopman, 2011; Mclaughlin, 2009; Mol, 2011; Mol et al., 2010; Nettleton, 2013). A market logic implies that customers have the possibility and obligation to rationally pick and choose services to meet their own needs (Mclaughlin, 2009); it entails that they have purchasing power (Mol et al., 2010). However, our results indicate that the notion of patients as customers in the healthcare context does not sufficiently capture what counts as being a person seeking care for bodily sensations and symptoms when suspecting cancer. Instead, people experience the contradiction in response to conflicting norms, by having to act as both a *selle*r and a *customer* during encounters with PCPs.

Our first theme captures attributes of a *seller* since it illuminates patients’ responsibilities of presenting and/or “selling” their perceived bodily sensations and symptoms to their PCP. The challenges are made visible by the fact that our study participants voiced concerns with “presenting the right” information to their PCP, to enable the PCP to really understand their reasons for care seeking, as well as the perceived need to be effective and seize the opportunity during the encounter, and not waste unnecessary time. Consequently, to describe patients as customers is not enough to capture all layers of what counts as being a patient, actually, it is quite problematic. In comparison with a customer on the market, a patient in the healthcare context does not possess the power to freely pick and choose which healthcare services they want to purchase. Rather, the patient is dependent on the care provider, here a PCP who “buys” services on behalf of the customer (patient) (e.g., Nettleton, 2013).

Our findings suggest that the market logic underlying standardization of care (CPPs) does not work optimally, since patients’ presentations of experienced bodily sensations and symptoms are impossible to standardize. As described by Mol (2011), the logic of care is defined as an open and interactive process that is shaped and re-shaped by the individual needs of the patient; it is not limited to a product nor to time. In contrast, the market logic is described as an arena where transactions are made and product matched with potential customers, thereby entailing the existence of purchasing power (Mol, 2011; Mol et al., 2010). We argue that the logic behind CPPs is similar to the market logic, namely, matching the CPP “product” with potential needs of “customers” (patients’ symptoms). However, it is not working as smoothly as it is expected to do, since interpretation of bodily changes is much more complex and difficult to standardize than needs and services in a market logic.

Hence, the contradiction of being both *seller* and *customer* is even evident in our results. Consequently, the “products” they wanted to consume or “buy”, were referrals to healthcare services from specialist care. However, to be eligible such “purchase”, they first have to “sell” their reasons for it, in other words, communicate their bodily sensations and symptoms to their PCP in an assuring way. Also, if presented symptoms match alarm symptoms conveyed in the CPPs, these symptoms qualify patients for the next level of services, such as a fast track. Consequently, CPPs may aggravate the challenges of presenting the sensations and symptoms, since PCPs are indirectly supposed to interpret symptoms through the lens of CPPs. However, when patients succeeded in “selling” their reasons and getting a referral into a fast track route, i.e., CPP, study participants described feeling taken care of. This could be interpreted as a satisfied customer that has the product they were looking for. Those patients who were referred into a CPP in our study, their experiences and appreciation of the fast track system are in line with previous research findings regarding CPPs, which indicate an increased patient satisfaction, and a more positive overall experience of the pre-diagnosis phase, possibly due to shorter time to diagnosis (Dahl et al., 2017; Sandager et al., 2019). On the contrary, those not referred into a CPP in our study perceived that the information they received was sometimes insufficient, which may contribute to feelings of uncertainty and anxiety. However, knowing that there is a way forward and a plan for their next steps in their trajectory was expressed as reassuring. Additionally, people who seek care, thus seeking to enter the ´sick role´ (Parsons, 1964), and want a referral to secondary care, are sometimes denied (Freund, 2003). The ´sick role´ is a position over which physicians have power because they have the ability to legitimize the patient’s presented illness experiences (Mik‐Meyer & Obling, 2012); thus, it is not free for the patient to “buy” the care they want. Our findings indicate that the power is not solely in the possession of PCPs, rather their power is mediated by CPPs, which previous research also suggests (Hultstrand, et al., 2020b). This reflects a market logic from a top-to-bottom perspective.

Our findings indicate that increased use of standardized routines in healthcare, and especially primary care, brings the market logic into the logic of care. As Andersen et al. suggest, the strong focus on efficiency can make the encounter merely an arena for clinically relevant exchanges, since it might hinder people from feeling comfortable to present vague symptoms and uncertainties (Andersen et al., 2015). Portraying people who seek care as customers might also impede a fruitful compassionate encounter, making the interaction more businesslike (Goldstein & Bowers, 2015). Hence, the ideal of increasing the efficiency in healthcare, by means of standardization, might impede a beneficial patient–provider interaction. Consequently, it is necessary to acknowledge that caring practices are more than just transactions.

According to our results, it is more challenging to present and verbalize diffuse or vague bodily sensations to a PCP than presenting more straightforward ones. As Andersen and Offersen with colleagues argue, medicine knowledge, such as alarm symptoms, might increase uncertainties (Andersen et al., 2015; Offersen et al., 2018;) even though the intention is the opposite. This is because the current discourse of early detection of cancer constructs alarm symptoms as black and white and denotes that cancer can be identified by being attentive to these early signs, when in reality, what people experience, is rather fluctuated, nuanced and dissolves in aspects of everyday life (Andersen, 2017; Offersen et al., 2018). Consequently, presenting vague and diffuse sensations and symptoms is often challenging to legitimate (Andersen et al., 2015), which participants in our study also confirmed. Thus, we interpret our findings to indicate that patients take the role as a *seller* during the encounter in order to “sell” their sensations and symptoms to their PCP, and consequently balance the contradiction of being both a *seller* and a *customer*.

Additionally, the foci on early detection and symptom awareness encompasses an ambivalence in terms of being aware of these alarm symptoms, being attentive towards bodily changes but not to exaggerated levels, as well as seeking care at a proper minute (Offersen et al., 2018; Ziebland et al., 2019). Besides, we do know that alarm symptoms function as something for PCPs to be attentive to and that alarm symptoms have distinct roles in the standardized routines (i.e. CPPs). Thereby, CPPs as a logic may complicate management of these conflicting norms patients face when seeking care. Furthermore, as so well put by Andersen et al. (2015), presenting with a chief complaint, rather than with vague and diffuse symptoms, encompasses a new level of what counts as being “a good patient”.

In sum, patients experience demands on themselves related to their care seeking, and they enter primary care with certain expectations regarding their care trajectories. Thus, patients juggle the twofold role of seller and customer when interacting with primary care.

### Strengths and weaknesses of the study

One strength with this interview study is that it is succeeding from a participant observational study (Hultstrand, et al., 2020a). That means that the participants have had their encounter observed and were thus familiar with the researcher prior to their interview. This might have favoured the interview since participants hopefully felt more relaxed with the interviewer. Second, triangulation in terms of multiple researchers can deepen the understanding of a studied phenomenon (Malterud, 2001); hence, we consider the diversity of the authors’ academic and professional backgrounds as a strength of this study. However, our study has some limitations that merit comments. First, when conducting interview studies, there is a potential risk of recall bias (Yin, 2009). However, the majority of the interviews were conducted in close time to their encounter; consequently, we assume that the risk of recall bias is reduced. Also, seeking care is a practice beyond the daily life routines with emotional attachment, for most of us, therefore we assume that such practices will be easier to recall. Second, some of the interviews were rather short, especially one that only lasted for 15 minutes. This could of course be seen as a limitation of the study. However, since the interviewee and the interviewer were familiar (due to the previously conducted observations) with each other the interviews begun smoothly and directly on the topic. Furthermore, the interviewees knew in prior to the interviews the topic of the interviews, and the questions were directly link to a single episode (their primary care encounter) which allowed the interviewees to easily respond. Thirdly, only four patients in our sample were referred into a CPP, meaning that the first part of our third theme is based on rather scares number of voices. However, these voiced experiences provide an insight of what it means, for these persons, to be referred into this fast track route, nevertheless, further research is needed.

### Conclusion

The conflicting norms development within the healthcare system put much responsibility and demands on the patients to act as good patients who recognize symptoms and seek care timely without wasting PCP’s time. This entails that patients juggle the roles of being both a *seller* and a *customer* in order to legitimize their care seeking, to get the care they need and expect. Patients seem to be aware of the complexity of the twofold role as both a *seller* and *customer*, hence, they invest much resources in managing the ambivalences and conflicting norms embedded in the logics of today’s healthcare. The idea of making healthcare more effective by implementing the logic of market into the logic of care seems to be a way to simplify a complex knowledge and understanding of human illness and healthcare seeking practices. Thus, standardization with its roots in a market logic is not that easy to apply in healthcare, since the logic of care is more than a transaction of products and is thus impossible to standardize. Consequently, presenting with symptoms that do not hold a certain standard (e.g., symptoms that are vague and diffuse) might increase patients’ ambivalence of their healthcare seeking practices.

## Clinical implications

This study illuminates that standardization oversimplifies the complexity underlying patients' experience of care seeking and interaction with healthcare and neglects the individual variation of each person's situation and needs. Hence, even if healthcare is standardized, patients’ needs of information and explanations concerning what can or will happen, and why, must be acknowledged by healthcare professionals.

## References

[cit0001] Andersen, R. S., Tørring, M. L., & Vedsted, P. (2015). Global health care–seeking discourses facing local clinical realities: Exploring the case of cancer. *Medical Anthropology Quarterly*, 29(2), 237–13. 10.1111/maq.12148 25355457

[cit0002] Andersen, R. S., Vedsted, P., Olesen, F., Bro, F., & Søndergaard, J. (2011). Does the organizational structure of health care systems influence care-seeking decisions? A qualitative analysis of Danish cancer patients’ reflections on care-seeking. *Scandinavian Journal of Primary Health Care*, 29(3), 144–149. 10.3109/02813432.2011.585799 21861597 PMC3347951

[cit0003] Andersen, R. S. (2017). Directing the senses in the contemporary orientations to cancer disease control: Debating symptom research. *Tidsskrift for Forskning i Sygdom og Samfund*, 14(26), 145–12. 10.7146/tfss.v14i26.26282

[cit0004] Boyer, C. A., & Lutfey, K. E. (2010). Examining critical health policy issues within and beyond the clinical encounter: Patient-provider relationships and help-seeking behaviors. *Journal of Health and Social Behavior*, 51(1_suppl), S80–S93. 10.1177/0022146510383489 20943585

[cit0005] Braun, V., & Clarke, V. (2006). Using thematic analysis in psychology. *Qualitative Research in Psychology*, 3(2), 77–101. 10.1191/1478088706qp063oa

[cit0006] Burr, V. (2015). *Social constructionism*. Routledge.

[cit0007] Clarke, V., & Braun, V. (2017). Thematic analysis. *The Journal of Positive Psychology*, 12(3), 297–298. 10.1080/17439760.2016.1262613

[cit0008] Dahl, T. L., Vedsted, P., & Jensen, H. (2017). The effect of standardised cancer pathways on Danish cancer patients’ dissatisfaction with waiting time. *Danish Medical Journal*, 64 (1) , 1. 28007051

[cit0009] Ewing, M., Naredi, P., Zhang, C., & Månsson, J. (2016). Identification of patients with non-metastatic colorectal cancer in primary care: A case-control study. *The British Journal of General Practice: The Journal of the Royal College of General Practitioners*, 66(653), e880. 10.3399/bjgp16X687985 27821670 PMC5198653

[cit0010] Freund, P. E. S. (2003). *Health, illness, and the social body: A critical sociology*. Prentice Hall.

[cit0011] Goldstein, M. M., & Bowers, D. G. (2015). The patient as consumer: Empowerment or commodification? Currents in contemporary bioethics. *The Journal of Law, Medicine & Ethics*, 43(1), 162–165. 10.1111/jlme.12203 25846046

[cit0012] Hajdarevic, S. 2012. *Patient and health care delays in malignant melanoma* [Dissertation]. Umeå University.

[cit0013] Hajdarevic, S., Hörnsten, Å., Sundbom, E., Brulin, C., & Schmitt-Egenolf, M. (2010). Patients’ decision making in seeking care for suspected malignant melanoma: Patients’ decision making in seeking care for malignant melanoma. *Journal of Nursing and Healthcare of Chronic Illness*, 2(2), 164–173. 10.1111/j.1752-9824.2010.01057.x

[cit0014] Hajdarevic, S., Schmitt‐Egenolf, M., Brulin, C., Sundbom, E., & Hörnsten, Å. (2011). Malignant melanoma: Gender patterns in care seeking for suspect marks. *Journal of Clinical Nursing*, 20(17–18), 2676–2684. 10.1111/j.1365-2702.2011.03788.x 21777314

[cit0015] Hartzband, P., & Groopman, J. (2011). The new language of medicine. *The New England Journal of Medicine*, 365(15), 1372–1373. 10.1056/NEJMp1107278 21995384

[cit0016] Healt and Medical Act 2017:80. Retrieved June 24, 2020, from https://www.riksdagen.se/sv/dokument-lagar/dokument/svensk-forfattningssamling/halso–och-sjukvardsforordning-201780_sfs-2017-80

[cit0017] Hultstrand, C., Coe, A. B., Lilja, M., & Hajdarevic, S. (2020b). GPs’ perspectives of the patient encounter - in the context of standardized cancer patient pathways. *Scandinavian Journal of Primary Health Care* 38 (2) , 1–10 doi:10.1080/02813432.2020.1753388. 32314634 PMC8570742

[cit0018] Hultstrand, C., Coe, A.-B., Lilja, M., & Hajdarevic, S. (2020a). Negotiating bodily sensations between patients and GPs in the context of standardized cancer patient pathways – An observational study in primary care. *BMC Health Services Research* 20 (46) , 1–12 doi:10.1186/s12913-020-4893-4. PMC6969453 31952534

[cit0019] Ingebrigtsen, S. G., Scheel, B. I., Hart, B., Thorsen, T., & Holtedahl, K. (2013). Frequency of ‘warning signs of cancer’ in Norwegian general practice, with prospective recording of subsequent cancer. *Family Practice*, 30(2), 153. 10.1093/fampra/cms065 23097250

[cit0020] Ingeman, M. L., Christensen, M. B., Bro, F., Knudsen, S. T., & Vedsted, P. (2015). The Danish cancer pathway for patients with serious non-specific symptoms and signs of cancer-a cross-sectional study of patient characteristics and cancer probability. *BMC Cancer* 15 (1) , 1–11. 10.1186/s12885-015-1424-5 25990247 PMC4445271

[cit0021] Jutel, A. (2011). *Putting a name to it: Diagnosis in contemporary society*. Johns Hopkins University Press. 10.7748/en.19.8.11.s7 27646036

[cit0022] Kleinman, A. (1980). *Patients and healers in the context of culture: An exploration of the borderland between anthropology, medicine, and psychiatry* (California: University of California press).

[cit0023] Lyratzopoulos, G., Vedsted, P., & Singh, H. (2015). Understanding missed opportunities for more timely diagnosis of cancer in symptomatic patients after presentation. *British Journal of Cancer*, 112(S1), 1. 10.1038/bjc.2015.47 25734393 PMC4385981

[cit0024] Macartney, J., Malmström, M., Nielsen, T., Evans, J., Hajdarevic, S., Chapple, A., …, and Ziebland, S. (2017). Patients’ initial steps to cancer diagnosis in Denmark, England and Sweden: What can a qualitative, cross-country comparison of narrative interviews tell us about potentially modifiable factors? *BMJ Open* 7 (11) , 7. 10.1136/bmjopen-2017-018210 PMC5702025 29151441

[cit0025] Malterud, K. (2001). Qualitative research: Standards, challenges, and guidelines. *The Lancet*, 358(9280), 483–488. 10.1016/S0140-6736(01)05627-6 11513933

[cit0026] Mclaughlin, H. (2009). What’s in a Name: ‘Client’, ‘Patient’, ‘Customer’, ‘Consumer’, ‘Expert by Experience’, ‘Service User’—What’s Next? *British Journal of Social Work*, 39(6), 1101–1117. 10.1093/bjsw/bcm155

[cit0027] Michailakis, D., & Schirmer, W. (2010). Agents of their health? How the Swedish welfare state introduces expectations of individual responsibility. *Sociology of Health & Illness*, 32(6), 930–947. 10.1111/j.1467-9566.2010.01262.x 20649889

[cit0028] Mik‐Meyer, N., & Obling, A. R. (2012). The negotiation of the sick role: General practitioners’ classification of patients with medically unexplained symptoms. *Sociology of Health & Illness*, 34 (7) , 1025–1038. 10.1111/j.1467-9566.2011.01448.x 22384857 PMC3555361

[cit0029] Mol, A., Moser, I., & Pols, J. E. (2010). *Care in practice: On tinkering in clinics, homes and farms*. Transcript.

[cit0030] Mol, A. (2011). *Omsorgens logik: aktiva patienter och valfrihetens gränser, Lund, Arkiv. English version: The logic of care: Health and the problem of patient choice. 2008*. Routledge.

[cit0031] Nettleton, S. (2013). *The sociology of health and illness*. Polity.

[cit0032] Nordgren, L. (2008). The performativity of the service management discourse - “Value creating customers” in health care. *Journal of Health Organization and Management*, 22(5), 510–528. 10.1108/14777260810898723 18959302

[cit0033] Offersen, S. M. H., Risør, M. B., Vedsted, P., & Andersen, R. S. (2016). Am I fine? Exploring everyday life ambiguities and potentialities of embodied sensations in a Danish middle-class community. *Medicine Anthropology Theory*, 3 (3) , 23–45. 10.17157/mat.3.3.392

[cit0034] Offersen, S. M., Risør, M., Vedsted, P., & Andersen, R. (2018). Cancer-before-cancer: Mythologies of cancer in everyday life Medicine Anthropology Theory - An open-access journal in the anthropology of health, illness, and medicine . 5((5) 30–52). 10.17157/mat.5.5.540.

[cit0035] Parsons, T. (1964). *The social system*. The Free Press.

[cit0036] Quaife, S. L., Forbes, L. J. L., Ramirez, A. J., Brain, K. E., Donnelly, C., Simon, A. E., & Wardle, J. (2013). Recognition of cancer warning signs and anticipated delay in help-seeking in a population sample of adults in the UK. *British Journal of Cancer*, 110(1), 12. 10.1038/bjc.2013.684 24178761 PMC3887291

[cit0037] Regional Cancer Center. *Standardized cancer patient pathway colorectal cancer*. Retrieved June 25, 2020, from https://kunskapsbanken.cancercentrum.se/diagnoser/tjock-och-andtarmscancer/vardforlopp/#-Utredning-och-beslut-om-behandling

[cit0038] Robb, K., Stubbings, S., Ramirez, A., Macleod, U., Austoker, J., Waller, J., Hiom, S., & Wardle, J. (2009). Public awareness of cancer in Britain: A population-based survey of adults. *British Journal of Cancer*, 101(S2), S18. 10.1038/sj.bjc.6605386 PMC2790705 19956158

[cit0039] Rubin, G., Berendsen, A., Crawford, S. M., Dommett, R., Earle, C., Emery, J., & Zimmermann, C. (2015). The expanding role of primary care in cancer control. *The Lancet Oncology*, 16 (12) , 1231–1272. 10.1016/S1470-2045(15)00205-3) 26431866

[cit0040] Sandager, M., Jensen, H., Lipczak, H., Sperling, C. D., & Vedsted, P. (2019). Cancer patients’ experiences with urgent referrals to cancer patient pathways. *European Journal of Cancer Care*, 28(1), e12927. 10.1111/ecc.12927 30303244

[cit0041] Seibaek, L., Petersen, L. K., Blaakaer, J., & Hounsgaard, L. (2011). Symptom interpretation and health care seeking in ovarian cancer. *BMC Women’s Health*, 11(1), 31. 10.1186/1472-6874-11-31 21699682 PMC3135550

[cit0042] Simon, A. E., Waller, J., Robb, K., & Wardle, J. (2010). Patient delay in presentation of possible cancer symptoms: The contribution of knowledge and attitudes in a population sample from the UK. *Cancer Epidemiology, Biomarkers & Prevention: A Publication of the American Association for Cancer Research, Cosponsored by the American Society of Preventive Oncology*, 19(9), 2272. 10.1158/1055-9965.EPI-10-0219 PMC2938472 20660602

[cit0043] Singh, H., Giardina, T. D., Meyer, A. N. D., Forjuoh, S. N., Reis, M. D., & Thomas, E. J. (2013). Types and origins of diagnostic errors in primary care settings. *JAMA Internal Medicine*, 173(6), 1–8. 10.1001/jamainternmed.2013.2777 23440149 PMC3690001

[cit0044] Whitaker, K. L., Macleod, U., Winstanley, K., Scott, S. E., & Wardle, J. (2015). Help seeking for cancer ‘alarm’ symptoms: A qualitative interview study of primary care patients in the UK. *The British Journal of General Practice: The Journal of the Royal College of General Practitioners*, 65(631), e96. 10.3399/bjgp15X683533 25624313 PMC4325458

[cit0045] Wilkens, J., Thulesius, H., Schmidt, I., & Carlsson, C. (2016). The 2015 national cancer program in Sweden: Introducing standardized care pathways in a decentralized system. *Health Policy*, 120(12), 1378–1382. 10.1016/j.healthpol.2016.09.008 27823827

[cit0046] Winstanley, K., Renzi, C., Smith, C. F., Wardle, J., & Whitaker, K. L. (2016). The impact of body vigilance on help-seeking for cancer ‘alarm’ symptoms: A community-based survey. (Report). *BMC Public Health*, 16((1) 1–6). 10.1186/s12889-016-3846-7. 27871273 PMC5117619

[cit0047] Yin, R. K. (2009). *Case study research: Design and methods*. SAGE.

[cit0048] Ziebland, R., Macartney, H., & Sand, A. (2019). How wide is the Goldilocks Zone in your health system? Journal of Health Services Research & Policy, 24, 52–56. 10.1177/1355819618790985 30060724

